# TRPV4 Channel in Neurological Disease: from Molecular Mechanisms to Therapeutic Potential

**DOI:** 10.1007/s12035-024-04518-5

**Published:** 2024-09-28

**Authors:** Feng Zhang, Hritik Mehta, Hadi Hasan Choudhary, Rezwanul Islam, Khalid A. Hanafy

**Affiliations:** 1 Cooper Medical School at Rowan University, Camden, NJ USA; 2 Cooper University Health Care, Camden, NJ USA; 3 Center for Neuroinflammation at Cooper Medical School at Rowan University, Camden, NJ USA; 4 Cooper Neurological Institute Center for Neuroinflammation, Cooper Medical School at Rowan University, Camden, NJ USA

**Keywords:** TRPV4, Modality-specific pharmacological agents, Neurological diseases, Microglia, Stroke

## Abstract

Transient Receptor Potential Vanilloid 4 (TRPV4) is a non-selective cation channel with pivotal roles in various physiological processes, including osmosensitivity, mechanosensation, neuronal development, vascular tone regulation, and bone homeostasis in human bodies. Recent studies have made significant progress in understanding the structure and functional role of TRPV4, shedding light on its involvement in pathological processes, particularly in the realm of neurological diseases. Here, we aim to provide a comprehensive exploration of the multifaceted contributions of TRPV4 to neurological diseases, spanning its intricate molecular mechanisms to its potential as a target for therapeutic interventions. We delve into the structural and functional attributes of TRPV4, scrutinize its expression profile, and elucidate the possible mechanisms through which it participates in the pathogenesis of neurological disorders. Furthermore, we discussed recent years’ progress in therapeutic strategies aimed at harnessing TRPV4 for the treatment of these diseases. These insights will provide a basis for understanding and designing modality-specific pharmacological agents to treat TRPV4-associated disorders.

## Introduction

Ion channels are membrane proteins that are involved in multiple cellular functions including the transport of electrolytes, generation of electrical signals, and secretion of hormones [1, 2]. The transient receptor potential (TRP) superfamily was initially cloned from fruit flies half a century ago, with subsequent genetic and electrophysiological investigations pinpointing these nonselective cation channels as the agents responsible for the depolarization of photoreceptor cells upon exposure to light [3–6]. Since then, over 30 subtypes of TRP channels have been found in mammals, encompassing a diverse range of ion channels with pivotal roles in sensory transduction, ion homeostasis, and cellular signaling [7–9]. More importantly, TRP channel mutants are tightly associated with numerous human disorders (like polycystic kidney disease, Night blindness, and familial episodic pain syndrome) [10–13]. TRPV4, among the various TRP channels, was initially discovered in the kidneys of rats and identified as a vertebrate homolog of the *Caenorhabditis elegans* gene Osm-9 [14], emerges as a key player in numerous pathological processes, including neuropathy, skeletal dysplasia, tumors, and inflammation [15–17]. Additionally, it plays a crucial role in physiological processes like osmosensation, mechanosensation, and thermosensation [18, 19]. With the advent of Cryo–Electron Microscopy, detailed atomic analysis is now unlocking the secrets of TRP channel subtypes, shedding light on the structural foundations of stimulus detection and gating, and deepening our understanding of how these channels are implicated in diseases linked to their function [20–25].

## The Basic of TRPV4

### Expression Profile of TRPV4

**Fig. 1:**
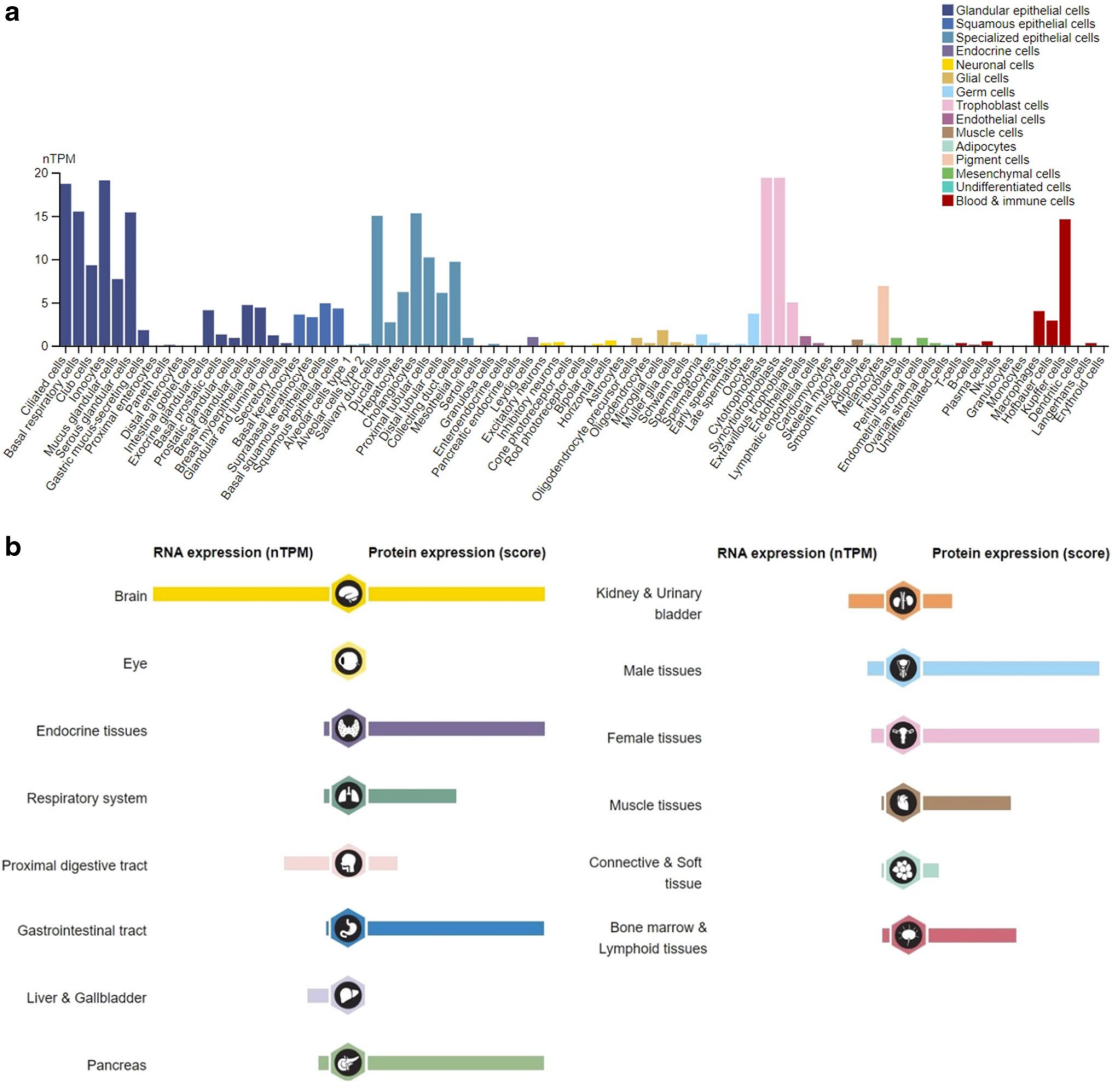
Microglia cells display the highest RNA expression levels among different cell types

TRPV4 is one of the most extensively investigated TRP channels due to its widespread expression and multifaceted roles across various systems, including the peripheral and central nervous systems, immune cells, cardiovascular system, urinary system, digestive tissues, and the respiratory tract [18, 19, 26]. On a single-cell level, the highest RNA expression is observed in ciliated cells, cytotrophoblast cells, and syncytiotrophoblasts. This expression pattern allows the coupling of TRPV4 with TMEM16F, a Ca^2+^-activated phospholipid scramblase (CaPLSase), during placental trophoblast syncytialization—a pivotal event in placental development [27]. In diverse tissues, monocytes and the cells derived from them consistently demonstrate elevated TRPV4 RNA expression levels. For example, in the liver, Kupffer cells exhibit the highest levels of TRPV4 RNA expression. A similar scenario is observed in the brain, where microglia cells display the highest RNA expression levels among different cell types (Fig. 1A). Notably, according to human protein atlas databank (https://www.proteinatlas.org/ ENSG00000111199-TRPV4), a significant disparity exists between TRPV4 RNA and protein expression levels, suggesting that TRPV4 expression is meticulously regulated at multiple levels within different physiological systems (Fig. 1, B). The impact of these expression levels on the progression of TRPV4- related diseases remains a topic of uncertainty, warranting further investigation. Another notable characteristic of TRPV4 expression lies in the distinct differences observed across various species. For instance, based on Brain RNA-seq databank (https://brainrnaseq.org/), in mouse brains, the RNA levels of TRPV4 expression are notably low in neurons, astrocytes, and oligodendrocytes. In contrast, in human brains, these same cell types, including neurons, oligodendrocytes, astrocytes, and endothelial cells, exhibit substantial higher RNA levels of TRPV4. Moreover, even macroglial cells express the highest RNA levels of TRPV4 both in humans and mouses. The extent to which these distinct expression patterns may influence experimental outcomes in mouse models remains largely uncertain, underscoring the importance of results obtained from studies using human tissues and cells.

### Structure Advances of TRPV4 Channels

#### TRPV Family and Structural Relationship

The structural elucidation of TRPV channels has undergone significant expansion over the past decade since the groundbreaking cryo-electron microscopy (cryo-EM) structure of TRPV1 was unveiled in 2013 [20]. Presently, over 200 structures with near-atomic resolution have been determined, spanning across more than 30 distinct TRP subfamilies [28, 29]. Like other established canonical TRP channels, most TRPV channels are structured as tetramers, each composed of six transmembrane domains (S1-S6) which forms a non-selective pore that allows the passage of Na + , K + , and Ca^2+^, except for TRPV5 and, which exhibiting much less sequence similarity and a strong preference for Ca^2+^ over Na + (over 100 times) [9, 30, 31]. Although there is minimal functional overlap, numerous molecular properties within the TRPV channel family are transferable among different members. For instance, with just several amino acid substitutions, the specificity of the TRPV1 agonist, resiniferatoxin, can be transferred to TRPV2 or TRPV3 [32–34]. Moreover, 2-Aminoethoxydiphenyl borate (2-APB), which serves as a universal agonist for TRPV1-3, but cannot activate wild type TRPV4, by replacing only two amino acids each at N- and C terminus, TRPV4 can be robustly activated by this compound [24, 35]. Another shared characteristic among classic TRPV channels (TRPV1-4) is their responsiveness to warm temperatures, each with distinct temperature thresholds, with only one or two-point mutations, these temperature thresholds can be altered drastically [36–38]. These findings collectively suggest due to high homology in protein sequences within the TRPV channel family, the gating mechanisms are highly conserved, which poses challenges in the development of highly specific agonists and antagonists for individual TRPV members.

#### Comprehensive Protein-Protein Interactions of TRPV4

**Fig. 2:**
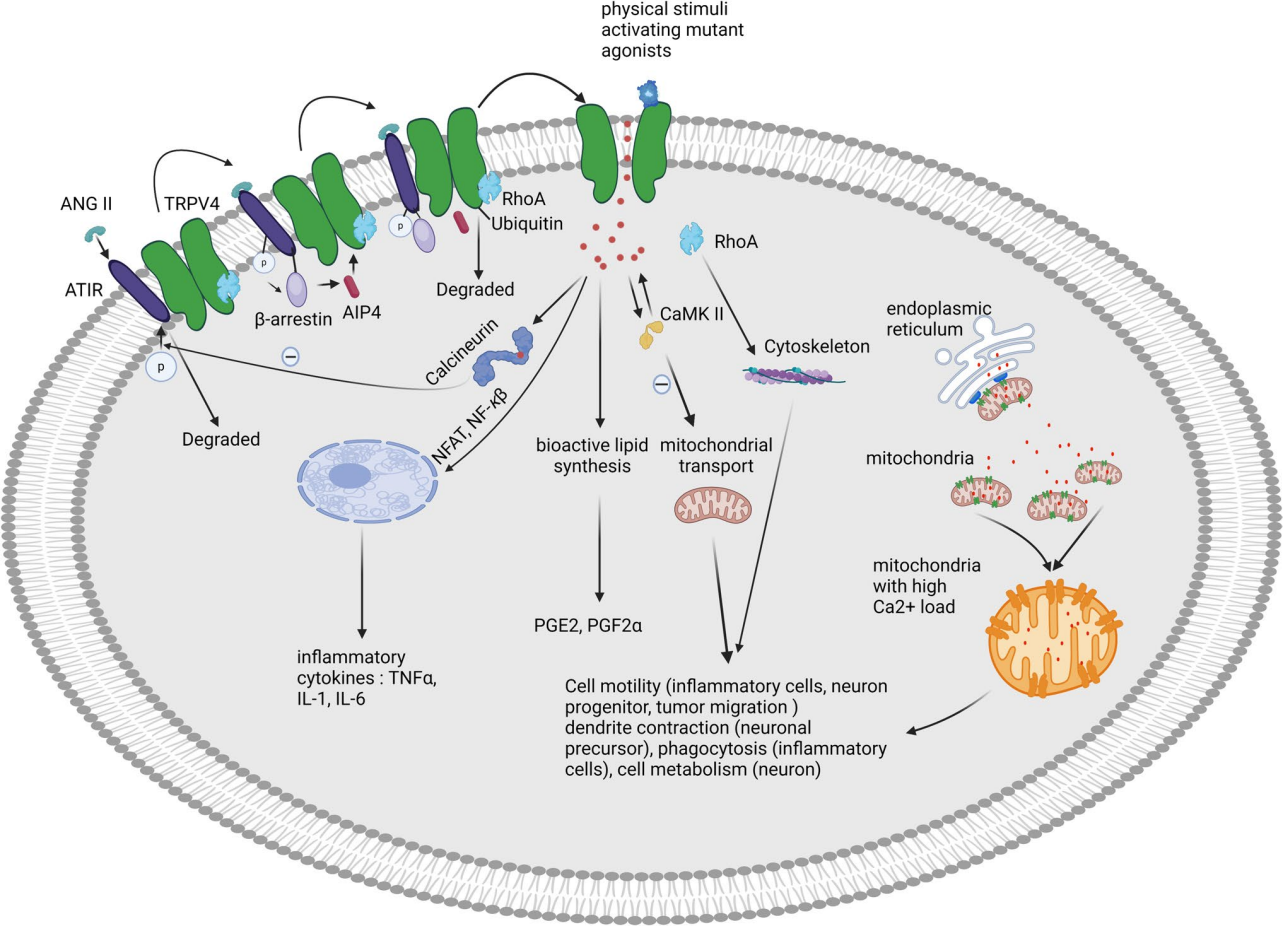
Cellular processes ultimately leading to cell contraction and a reduction in the extension of cellular processes and affect neurite growth

Over 20 cryoEM structures of full-length and truncated TRPV4 have been determined today, including those from humans and Xenopus tropicalis, with various agonists and antagonists bound [24, 39–42]. These structures have significantly enhanced our understanding of this important channel and its gating mechanisms. In Xenopus tropicalis TRPV4, Deng et al. reported a ligand-free, non-conducting state with an atypical non-domain-swapped tetrameric arrangement, raising questions about its physiological relevance, which may be due to the truncated version of the channel [39]. In contrast, recently published human TRPV4 structures (both full-length and truncated) by several groups consistently show a typical domain-swapped structure with overall minor differences. Structurally, TRPV channels are distinguished by their substantial N-ankyrin domain, which plays a pivotal role in gating these channels [20–22, 24, 43], of particular interest is the observation that most TRPV4 mutations associated with neuropathic diseases are located in the N-terminal domain, highlighting its importance in TRPV4’s function [24]. Another characteristic property of TRPV4 is its well-documented involvement in numerous protein complexes. As so far, at least 38 interacting proteins have been identified for TRPV4 [44], including various G protein-coupled receptors (GPCRs) [45, 46], GTPase, [24, 41, 47] and other ion channels such as TRPC1 and TRPP2 [48, 49]. Like TRP channels, GPCRs respond to extracellular stimuli and translate them into intracellular signaling, particularly in response to tissue damage or infection. GPCR activities are heavily dependent on various downstream signaling effectors, including guanine nucleotide exchange factors, kinases, phospholipases, adenylate cyclase, and ion channels [50–52]. Recent studies on the function of the TRPV4-GPCR complex have unveiled a direct interaction between these two essential proteins. This complex forms the foundation of TRPV4-related diseases and may shed light on TRPV4’s involvement in various conditions, including neuropathy [45, 46]. The intricate associations between TRPV4 and numerous diseases are rooted in these protein complexes, illustrating its far-reaching impact on biological processes. Our most recently published papers offer a clear depiction of the TRPV4-Rho complex structure, highlighting the pivotal role of TRPV4’s extended N- terminus in its interaction with RhoA [24]. The RhoA protein, a small GTPase, has been recognized as a key player in cytoskeletal remodeling, when in its active state, RhoA significantly promotes actin polymerization, stress fiber formation, and actomyosin contraction [47, 53]. These cellular processes ultimately lead to cell contraction and a reduction in the extension of cellular processes and affect neurite growth (Fig. 2).

## The Neurological-Related Diseases Related to TRPV4 and Its Mutations

The TRPV4 mutation has long been linked to a range of diseases, including peripheral neuropathies, skeletal dysplasia, and arthropathy, as evidenced by genome analysis [16, 26, 54–56]. However, recent years of research have increasingly demonstrated associations between this channel and a broader spectrum of diseases across various systems in the human body [17, 57–61].

### Peripheral Neuropathies and Skeletal Disorders

The most studied TRPV4 mutant-related heritable diseases are generally classified into two major categories: peripheral neuropathies and skeletal dysplasia [62]. The TRPV4-related peripheral neuropathies can be primarily categorized into three groups: Charcot-Marie-Tooth disease (CMT) type 2C, scapuloperoneal spinal muscular atrophy, and distal hereditary SMA (CDSMA) [63]. It is widely accepted that peripheral nerve degeneration linked to TRPV4 mutations in these disorders leads to muscle weakness, particularly affecting the limbs, diaphragm, and vocal fold muscles, posing potentially life-threatening implications. These mutations are dominantly inherited missense mutations, the severity of these disorders exhibits a striking variability, encompassing severe, congenital onset cases to milder, late adult-onset cases, even within the same family [64–66], which may suggest the existence of several layers of expression control for this channel, allowing it to adjust its function in response to changing characteristics, and it might indicate the presence of compensatory mechanisms aimed at mitigating the effects of malfunctioning proteins. TRPV4-mediated skeletal dysplasia and arthropathy represent another major TRPV4-related disorders which comprising over hundreds of disorders, often linked to orthopedic complications. In recent years, several mutations in the TRPV4 gene have emerged as an important contributor to various forms of dwarfism with a short trunk and a condition known as Familial digital arthropathy-brachydactyly (FDAB) [67–69]. Since there are numerous excellent reviews that delve into this aspect [11, 26, 70], we will not discuss this further in the review.

### Inflammation

Due to its widespread expression, polymodal activation, and calcium permeability, TRPV4 is crucial for modulating the function of the nervous system. Recent studies have shown that hyperactivation of TRPV4 leads to neurotoxicity, contributing to neuronal damage associated with conditions such as intracerebral hemorrhage, traumatic brain injury, and cerebral ischemia [71]. Considerable evidence supports the notion that TRPV4 activation triggers the release of proinflammatory cytokines, implicating its involvement in inflammation across various bodily systems such as the lung, gastrointestinal tract, adipose tissue, and retina. Within the central nervous system (CNS), neuroinflammation emerges as a significant contributor to neuronal injury in pathological conditions [61, 72–74]. TRPV4 exhibits high expression in monocytes cells and their derived cells like macrophages, kupffer cells etc., making it a possible candidate for involvement in inflammatory processes [17, 46, 75]. Known for its calcium-permeable properties, TRPV4 enable the influx of considerable amounts of Ca^2+^ into the cell [14], which in turn activates nuclear factor of activated T cells (NFAT) through Ca^2+^-dependent calcineurin, further upregulating inflammatory cytokines such as TNFα, IL-1, and IL-6, thereby linking it to several pathologically inflammatory conditions (Fig. 2), uncontrolled inflammation can exacerbate normal tissue damage while combating with the initial factors, such as microorganisms [76]. Therefore, maintaining tight regulation of TRPV4 in macrophage cells become a crucial strategy for the treatment of diseases associated with inflammation [77]. Recent studies also have indicated the critical role of TRPV4 in lung edema, especially in response to triggers such as Lipopolysaccharide (LPS)-induced acute lung injury [45, 60, 61, 76, 78]. Given the abundance of TRPV4 in endothelial cells and pulmonary epithelial cells, together with the high expression in macrophages, its significance in the initiation and maintenance of inflammation can be easily understood. Study evidence supporting the use of TRPV4 inhibition as a therapeutic target for such conditions is steadily growing [77, 79, 80].

### Alzheimer’s Disease (AD)

In the nervous system, TRPV4 displays a comprehensive expression pattern within the brain, covering cortex and hippocampus which are crucial regions in AD pathology, as well as presenting in diverse cell types, including neurons and microglia [54, 81]. Notably, in the context of Alzheimer’s disease, research conducted by Du and colleagues revealed that the activation of TRPV4 led to an increase in tau phosphorylation within the cortex and hippocampus of the P301S tauopathy mouse model, exacerbating cognitive decline [74]. Additionally, investigations have proposed that TRPV4 activation may disrupt cholesterol homeostasis in primary neurons, thereby promoting hyper-phosphorylation of tau. In mouse models with TRPV4 knockdown, reduced intracellular cholesterol accumulation resulted in the amelioration of tau hyper-phosphorylation [82].

As a calcium-permeable cation channel, TRPV4 may participate in neuronal death by various mechanisms including endothelial cells (ES) and vascular smooth muscle cells (SMC) damage. By mediating calcium influx from the extracellular fluid into the cytoplasm, which initiates a cascade of intracellular events, including an increase in mitochondrial calcium levels, and the release of cytochrome C, ultimately triggering apoptosis [83]. While the vascular aging has been vital to sporadic Alzheimer’s disease and has been under intensive investigation for years, studies, such as Norton et al., have observed that oxidative stress activates pathways beyond calcium signaling, contributing to cell death. In the context of posterior cerebral artery (PCAs) cells exposed to hydrogen peroxide (H2O2), the extent of SMC death is closely associated with an elevation in intracellular Ca^2+^ concentration. While TRPV4 is a key player in calcium influx, activation of TRPV4 channels alone does not induce cell death in either SMCs or ECs, suggesting the involvement of additional pathways in oxidative stress-induced cell death. In PCAs from young males, which are particularly susceptible to H2O2-induced damage, inhibiting TRPV4 channels significantly reduces SMC death and intracellular Ca^2+^ responses. However, in vessels from old or female mice, inhibition of TRPV4 channels has minimal effect on cell death and intracellular Ca^2+^ responses. These findings suggest that the role of TRPV4 channels in mediating H2O2-induced cell death varies depending on factors such as age and sex [83]. Collectively, these findings strongly suggest that the activation of TRPV4 may play an important role in the pathogenesis and progression of Alzheimer’s disease.

### Stroke

The disruption of the blood–brain barrier (BBB) and the subsequent development of brain edema plays critical roles in neuronal death and neurological dysfunction in stroke. The vascular endothelial cells within the brain, crucial for maintaining the BBB’s integrity, abundantly express calcium-permeable channel TRPV4. After a stroke, the activation of TRPV4 may occur in response to heightened intracranial pressure. This activation then initiates endothelial cell activation and contraction by rapidly inducing actin polymerization in endothelial cells. This process culminates in the formation of force-generating structures, ultimately resulting in an elevated permeability of the BBB, as Zhao et al. reported [84]. Targeting TRPV4 becomes a logical approach to break this detrimental cycle, positioning it as a natural regulator of BBB permeability. Strategies aimed at mitigating BBB disruption present a promising avenue for managing intracerebral hemorrhage (ICH). Given TRPV4’s abundant expression in endothelial and microglial cells, crucial components in the neuroinflammation process, TRPV4 inhibition offers multiple checkpoints to control stroke-induced neuroinflammation, targeting TRPV4 emerges as a promising therapeutic avenue to safeguard the BBB and modulate neuroinflammation, with implications for innovative strategies in managing these complex neurological conditions.

### Autism

Autism spectrum disorder (ASD) presents as a multifaceted neurodevelopmental condition characterized by difficulties in social interaction, communication, and the presence of restricted, repetitive behaviors [85]. Despite extensive research efforts, the precise etiology of autism remains elusive, prompting investigations into a myriad of genetic, environmental, and neurological factors that may contribute to its onset. Recently, studies have turned to the TRPV4 channel as a potential player in the pathophysiology of autism. It was reported that whole-genome sequencing of 85 quartet families (two parents and two ASD-affected children) with autism revealed the occurrence of frame-shift mutations in the TRPV4 gene among the autism patients [86]. Studies utilizing animal models have underscored the significance of TRPV4 in fundamental processes such as neuronal development, synaptic plasticity, and neurotransmitter release [47], all of which are mechanisms potentially dysregulated in autism. In a notable study by Stamatina Tzanoulinou et al., conducted in a mouse model of autism featuring knock-down in the Shank3 gene, it was observed that the overexpression of TRPV4 led to hyperexcitability in D1R-medium spiny neurons (D1R-MSNs) and impaired social behavior. Remarkably, the application of a TRPV4-specific inhibitor within the same brain region substantially restored typical social behaviors. These findings were corroborated in genetically susceptible Shank3 ± mice, wherein the induction of an acute inflammatory response via lipopolysaccharide resulted in analogous circuit and behavioral alterations, which were similarly rescued by TRPV4 inhibition [87]. Given the escalating prevalence of autism in recent years, these insights hold profound significance [88]. Although TRPV4 may represent just one downstream effector within a complex signaling pathway, its identification as a potential therapeutic target for this pervasive psychological disorder is compelling. To date, no instances of TRPV4 mutations directly linked to autism have been reported, underscoring the need for further investigation into its role in the disorder’s pathogenesis.

### Epilepsy

Epilepsy, a neurological disorder characterized by recurrent seizures, has complex etiologies involving various cellular and molecular mechanisms. The hallmark of epilepsy is abnormal neuronal firing. Recent evidence suggests a significant link between TRPV4 and epilepsy. This connection may involve astrocytes, as upregulated TRPV4 expression by Nuclear Factor I A (NFIA) leads to elevated calcium influx into astrocytes, triggers the release of pro-inflammatory cytokines and gliotransmitters, which enhance neuronal excitability and contribute to an epileptogenic state. Conversely, NFIA knockdown significantly inhibits 4-AP-induced TRPV4 expression and TRPV4-mediated calcium entry, thereby mitigating aberrant neuronal discharges, reducing neuronal damage, and suppressing epileptic seizures [89, 90].

Hyperthermia is a well-known seizure trigger, especially in individuals with epilepsy. TRPV4 is recognized as a temperature-sensitive channel, typically activated at around 27–34 °C. Studies have shown that physiological temperature activation of TRPV4 enhances neuronal excitability. Dynamic local temperature changes in the brain have been observed to correlate with neuronal bursts and EEG activity, suggesting that neuronal activity can partially evoke hyperthermia in vivo. In epileptogenic foci, local hyperthermia abnormally elevates neuronal activity via TRPV4 activation, creating a feedback loop where increased neuronal activity further raises the temperature. Cooling the brain reduces epileptic discharges more effectively in wild-type mice compared to TRPV4 knockout mice, indicating that TRPV4 plays a crucial role in the suppression of epileptic activity through brain cooling, and TRPV4-specific inhibitors effectively suppress epileptic discharges [89, 91]. Furthermore, blocking TRPV4 downregulates NF-κB signaling, reducing inflammation and neuronal death, which are critical in the progression of epilepsy [92]. Collectively, these mechanisms underscore the importance of TRPV4 in epilepsy, highlighting its potential as a therapeutic target. These findings have also been confirmed in zebrafish models, the activation of TRPV4 channels is crucial in the onset of hyperthermia-induced seizures and blocking these channels reduces seizure activity in zebrafish, highlighting their role in the mechanism [93], and this suggests that the role of TRPV4 in epilepsy may be a conserved mechanism across species.

## The Underlying Molecular Mechanism of TRPV4 in Nervous System

Unraveling a molecular mechanism’s link to a particular disease has always been a challenging endeavor. This difficulty stems from the intricate nature of biological systems, which are broadly interconnected within vast networks. Moreover, the human body boasts remarkable compensatory abilities, which can obscure the true underlying mechanisms when certain biological processes malfunction. This is especially complicated when considering TRPV4-related neuropathy and skeletal dysplasia show high variations between affected individuals even within the same family. As previously discussed, the fact that TRPV4 knock-out mice can thrive with minimal observable effects strongly suggests the existence of redundant mechanisms to compensate for the malfunctioning of mutated TRPV4 [57, 58, 94].

### TRPV4-GPCR Signaling Pathway

Over the past decades’ studies have established connections between TRPV4 and a diverse spectrum of conditions, including inflammation, peripheral neuropathy, skeletal dysplasia, vascular tone regulation, and tumor progression, among others. Considering TRPV4’s extensive structural and functional network, many of these diseases are associated with its pivotal role in specific signaling pathways. An illustrative instance of the TRPV4-GPCR axis represents a crucial pathway, substantiated by increasing evidence that underscores its significance in diverse pathological processes. For instance, the G protein-coupled receptor (PAR2) plays an important role in modulating inflammatory responses, obesity, and metabolism [95–100]. A recent study conducted by Rayees et al. has shed light on a novel mechanism wherein PAR2-mediated cAMP generation abolishes TRPV4-dependent Ca^2+^ signaling in alveolar macrophages, effectively resolving Toll-like receptor (TLR)-induced inflammation. The depletion of PAR2 through the use of small interfering RNA (siRNA), along with the consequent cAMP generation, amplifies Ca^2+^ entry via TRPV4. This, in turn, leads to sustained activation of the transcription factor NFAT, resulting in the prolonged manifestation of TLR4-mediated inflammatory lung injury. Notably, the inhibition of TRPV4 in PAR2 null mouse alveolar macrophages following the LPS challenge has been shown to promote inflammation resolution and facilitate the repair of lung injuries [99].

Furthermore, substantial evidence suggests a potential interaction between TRPV4 and angiotensin receptor II (AT1R). In vitro experiments have revealed that, upon AT1R activation, it initiates the recruitment of β-arrestin which in turn, associates with an E3 ubiquitin ligase responsible for catalyzing TRPV4 ubiquitination (Fig. 2) [45]. Consequently, this process results in a decrease in membranous TRPV4 levels. Conversely, when TRPV4 is activated by endogenous or synthetic agonists, it assumes the role of inhibiting AT1R/angiotensin II-mediated G-protein-associated second messenger accumulation, and β-arrestin recruitment by the calcium-activated phosphatase calcineurin. The inhibitory impact of β-arrestin-AIP4-ubiquitination on TRPV4 degradation potentially limits its capabilities of downregulating TRPV4 function, but TRPV4 activation concurrently impedes AT1R phosphorylation through calcium-activated phosphatase calcineurin in a Ca^2+^/calmodulin–dependent manner, hindering its internalization which may further limit excessive TRPV4 activity. This bidirectional regulation prompts a perplexing question regarding the predominant influence (Fig. 2). It likely operates as a protective mechanism, finely tuning TRPV4 activity to avert overactivation and maintain optimal intracellular calcium levels [46]. The intricate interplay within the TRPV4-AT1R complex may provide a potential explanation for the relatively normal blood pressure observed in individuals with peripheral neuropathy diseases resulting from gain-of-function mutations in TRPV4 since activating TRPV4 mutants may be inhibited by the abundant co-expression of AT1R in endothelial cells. While neurons lacking AT1R expression, particularly in those hosting gain-of-function TRPV4 variants, often exhibit dysregulated intracellular calcium levels [101]. This raises an intriguing query: could dysregulated intracellular calcium be a pivotal factor in neuropathy development? If this hypothesis is proven legitimate, it could pave the way for new genetic strategies in treating this debilitating disease, extending beyond mere TRPV4 inhibition.

### TRPV4 Induced Pro-inflammatory Protein Expression

As discussed earlier, the involvement of TRPV4 in inflammatory reactions has been observed in numerous laboratory studies. During the inflammation process, endothelial cells serve as pivotal effector cells. Elevated levels of intracellular Ca^2+^, facilitated by Ca^2+^ permeable channels like TRPV4, prompt rearrangements in the endothelial cell cytoskeleton. This, in turn, initiates inflammation and adjusts vascular tension, facilitating the migration of inflammatory cells such as macrophages across the blood vessel wall. However, there are studies have indicated that TRPV4 does not regulate microvascular endothelial permeability during brain inflammation [102]. It is widely acknowledged that TRPV4 contributes to inflammation through its Ca^2+^ permeable properties in both endothelial and macrophage cells, with Ca^2+^ playing a central role in the entire process. Intracellular Ca^2+^ levels are particularly significant in macrophage activation, as they govern a cascade of inflammatory gene expression, ultimately leading to a potent proinflammatory response. For example, Li et al. noted that inhibiting TRPV4 or using TRPV4 knockout cells could be reversed by LPS-induced intracellular Ca^2+^ elevation in macrophages and this reversal further led to a significant down-regulation in the expression of IL-6, ROS, and NFATc3 [76].

In mice models of Pilocarpine-Induced Status Epilepticus (PISE), An et al. demonstrated that TRPV4 antagonism significantly prolongs the latency for PISE development and reduces the success rate of PISE model preparation, suggesting TRPV4’s implication in epilepsy pathogenesis. The study revealed that TRPV4 activation increases the expression of HMGB1 (High mobility group protein B1) and TLR4, subsequently leading to phosphorylation of IκK and IκBα and resulting in NF-κB activation and nuclear translocation. This cascade ultimately enhances the production of pro-inflammatory cytokines, contributing to TRPV4 activation-induced neuronal injury. Conversely, TRPV4 blockade downregulates the NF-κB signaling pathway, suppressing inflammatory responses and the activation of cytoplasmic and nuclear p65 and p50. These findings offer evidence of anti-inflammatory response of TRPV4 and its neuroprotective capabilities in PISE [92].

### TRPV4 Gain-of-Function as Ca^2+^ Permeable Channel

From a functional perspective, the majority of TRPV4 mutations associated with disease exhibit activation (gain-of-function) characteristics. This suggests that TRPV4 mutants display heightened basal activities compared to wild-type TRPV4. Consequently, this leads to increased intracellular Ca^2+^ concentrations, offering significant insights into the molecular mechanisms driving the pathology of these diseases. In terms of morphological effects, neuropathy mutants of TRPV4 harboring neuronal cell line display impaired neurite length growth compared to transfected wild-type TRPV4 in MN-1 cells. When a TRPV4 inhibitor is introduced, it promotes neurite growth in both wild-type TRPV4 and neuropathy-causing mutants. The same phenomenon is also observed in skeletal dysplasia mutations like D333G, where TRPV4 antagonist GSK219 significantly boost neurite growth [47]. These findings underscore the significance of minimal TRPV4 activity in neurite extension, potentially offering insights into the development of inhibitors to treat diseases caused by gain-of-function TRPV4 mutations. Recent research has also demonstrated that the in vivo expression of a neuropathy-inducing TRPV4 mutant (TRPV4R269C) leads to neuronal dysfunction and axonal degeneration [101]. Strikingly, these detrimental effects can be reversed by employing genetic or pharmacological interventions that block TRPV4 channel activity. TRPV4 R269C initiates an increase in intracellular Ca^2+^ via a Ca^2+^/calmodulin-dependent protein kinase II (CaMKII)-mediated pathway, and the inhibition of CaMKII effectively counteracts this rise in intracellular Ca^2+^ and the associated neurotoxicity, as observed in both *Drosophila* and cultured primary mouse neurons. Furthermore, it is worth emphasizing that elevated TRPV4 activity disrupts axonal mitochondrial transport, and the neurotoxic impact of TRPV4 is intricately linked to the Ca^2+^-binding of mitochondrial GTPase. These findings underscore the critical role of elevated intraneuronal Ca^2+^ responses associated with increased basic TRPV4 activity, as well as the significance of tightly regulated Ca^2+^ dynamics in maintaining mitochondrial axonal transport [47, 101, 103, 104].

A noteworthy observation is that even though there are clinical cases showing mixed neuropathy and skeletal phenotypes due to TRPV4 gain-of-function mutant R616G [104], an intriguing observation emerges when examining different mutants linked to distinct disease types: mutations associated with skeletal diseases are found distributed across the entire protein length, those tied to peripheral neuropathies tend to concentrate on the N-terminus of the channel [26]. This suggests that while many TRPV4-related diseases result from channel gain-of-function, the underlying mechanisms vary significantly. Notably, in the context of skeletal dysplasia mutations such as D333G, the interaction between TRPV4 and RhoA appears to remain largely intact which suggests the gain-of-function of this mutant arises from its intrinsic structural rearrangement, implying a distinct role for TRPV4 itself and the associated elevation of intracellular Ca^2+^ levels in neuropathy rather than skeletal dysplasia [47]. However, it remains an enigma whether all identified neuropathy-causing mutants exhibit similar decreased TRPV4-Rho interaction pattern, and if proven true, this finding would substantially enhance our comprehension of the molecular mechanisms underlying TRPV4-related peripheral neuropathy.

### The TRPV4-RhoA Signaling Pathway

RhoA is well-known as a principal controller of cytoskeletal dynamics, functioning as a molecular switch that alternates between an inactive state bound to GDP and an active state bound to GTP. Mutations of RhoA have been linked to various cancers [105]. Recent structural insights have unequivocally shown that TRPV4 can form complexes with RhoA, and functional assays show this complex’s stability strongly hinges on TRPV4’s activation state [24, 41, 47]. During the transition of TRPV4 from an inhibitory state to an apo state and subsequently to an activated state, the electron density of RhoA decreases. During functional assays, gain-of-function mutants in the N-terminus also exhibit weaker interactions with TRPV4, a fact confirmed through in-vitro biochemical assays [24]. These findings suggest that RhoA has a greater propensity to bind to the inactivated or closed state of TRPV4, and further stabilize this conformation. On the other hand, activating mutants in TRPV4 may result from two mechanisms, one is that the mutant itself causes more simultaneous channel openings through its gating machinery; the other possibility is that its weakened binding with RhoA leads to shorter indwelling periods with RhoA, thus losing its ability to stabilize the closed TRPV4 state. The net effect of these mechanisms is the manifestation of gain-of-function properties by TRPV4.

The TRPV4-RhoA interaction signaling pathway introduces a novel perspective on the mechanisms underlying TRPV4-related diseases. Specifically, when RhoA is decoupled, acting as an inhibitor, it results in an increase in intracellular Ca^2+^ concentration. Conversely, in its decoupled (GTP-bound, activated) state, RhoA inhibits cytoskeletal outgrowth. Furthermore, inhibition of RhoA restores neurite length in vitro and in a fly model of TRPV4 neuropathy, as demonstrated in a fly model of TRPV4 R269C mutant, which mimics TRPV4 neuropathy. In this model, the restoration of neurite length in vitro through the inhibition of RhoA provides further compelling evidence supporting the potential therapeutic avenue of targeting TRPV4 to address the devastating diseases triggered by TRPV4 gain-of-function mutations [47, 103].

Even though the inhibitory neurite growth can explain the mechanism of TRPV4-related neuropathy, it is essential to recognize that other mechanisms may also contribute to TRPV4-related neuropathy. For example, recent findings show that TRPV4 plays a key role in tumor cell dissemination from three-dimensional tumor spheroids. The heightened microenvironmental viscosity amplifies the motility of various cell types on both two-dimensional surfaces and three-dimensional tumor spheroids through the activation of an actin-related protein 2/3 (ARP2/3) network. This activation ultimately triggers TRPV4 and subsequent calcium influx, leading to increased RhoA-dependent cell contractility [106]. The potential impact of TRPV4-regulated mobility on neuron precursor cells during nervous system evolution remains uncertain. In cases involving neuropathy-causing TRPV4 mutants, the potential reduction in interaction between RhoA and TRPV4 could potentially influence RhoA involved migration process. This could further decrease the number of motor neurons in the ventral horn during nervous system development, representing an intriguing area for further exploration in this field.

### TRPV4 and Mitochondrial Function

Mitochondria, as the cell’s powerhouses, play a critical role in the pathogenesis of neurodegenerative diseases such as Alzheimer’s, Huntington’s, and Parkinson’s [107–109]. Recent research has highlighted TRPV4-mediated mitochondrial dysfunction across various diseases, shedding light on potential mechanisms relevant to nervous system disorders. For instance, in osteoarthritis, TRPV4-induced.

calcium dysregulation disrupts mitochondrial dynamics via the Drp1-HK2 axis, leading to pyroptosis and cartilage degradation [110]. This pathway underscores TRPV4’s ability to exacerbate cellular stress and tissue damage by affecting mitochondrial function. Similarly, in primary murine T cells, TRPV4 modulates mitochondrial calcium levels based on their immunological state, influencing cellular physiology and immune [111]. These insights give us a hint that TRPV4-mediated mechanisms implicated in neuropathy such as CMT and Alzheimer’s, where mitochondrial dysfunction is pivotal, could contribute to neuronal degeneration and dysfunction. Studies elucidating the AKT/alpha-synuclein pathway in dopaminergic neurons further explore how TRPV4 dysregulation impacts mitochondrial function and neuronal survival in neurodegenerative conditions. In this study, Sun et al. discovered that neurons harboring the constitutively activating TRPV4 mutant L619F exhibited elevated cytosolic Ca^2+^ levels; this increase enhanced AKT-mediated induction of α-synuclein (α-syn), leading to mitochondrial Ca^2+^ accumulation and dysfunction. Notably, treatment with a TRPV4 antagonist, AKT inhibitor, or α-syn knockdown normalized mitochondrial Ca^2+^ levels in the mutant neurons [112]. This underscores the significance of the TRPV4/Ca^2+^/AKT-induced α-syn pathway in mitochondrial Ca^2+^ accumulation. Additionally, as discussed in the previous chapter, most, if not all, CMT-related TRPV4 mutants are gain-of-function mutations, and some of these mutants have been shown to enhance intracellular Ca^2+^, a mechanism similar to that of L619F. While in those studies, the focus was on how elevated intracellular Ca^2+^ leads to mitochondrial transport impairment and axonal degeneration [101], it is very likely that increased intracellular Ca^2+^ in these cells also contributes to elevated mitochondrial Ca^2+^ levels, further exacerbating neuropathy.

Numerous studies have established that excessive mitochondrial free reactive oxygen species (mitROS), induced by aging or acute hypoxia, play a crucial role in neuronal damage. TRPV4 is one of the Ca^2+^ channels activated by mitROS. Notably, amantadine, a drug that may offer protection for brain function, also acts as a TRPV4 antagonist. It attenuates oxidative neurotoxicity, mitochondrial membrane potential (ΔΨm) disruption, inflammation, and apoptosis in neuronal cells (SH-SY5Y) [113]. Similarly, as we have discussed, the inhibition of TRPV4 channels using HC-067047 effectively reduces Ca^2+^ entry and smooth muscle cell (SMC) death in response to H2O2 in posterior cerebral arteries (PCAs) from young males [83].

While the mechanisms linking TRPV4 to mitochondrial dysfunction may be multifaceted, emerging evidence challenges the traditional view of TRPV4 as solely a plasma membrane protein. It highlights TRPV4’s role as a mitochondrial Ca^2+^ importer, which is crucial for regulating mitochondrial temperature.

and metabolism [114]. This Ca^2+^ influx via TRPV4 supports ATP production by mitochondrial enzymes, thus influencing the metabolic activity essential for neuronal function. Furthermore, Acharya et al. investigated TRPV4’s interaction with mitofusin 2 (MFN2), which facilitates endoplasmic reticulum-mitochondrial contact points critical for Ca^2+^ buffering and transfer (Fig. 2). Disruption of these contact points can impair mitochondrial function and induce cellular stress, implicating TRPV4 in the pathophysiology of neurological disorders [115].

While current studies do not directly link mutated mitochondrial TRPV4 with neuropathy, understanding TRPV4’s impact on mitochondrial function offers new insights into the development of neurological disorders characterized by mitochondrial dysfunction affecting neurite extension. Further investigation into targeting TRPV4 for therapeutic interventions against neurological disorders associated with mitochondrial dysfunction holds potential for innovative treatments. This avenue of research warrants continued exploration to unravel the full therapeutic possibilities of TRPV4 modulation in mitigating neurological diseases linked to mitochondrial dysfunction.

## TRPV4 as a Therapeutic Target in Neurological Disease

The therapeutic potential of TRPV4 modulation is a rapidly evolving field due to the key role of TRPV4 in various diseases. We explore the molecular mechanisms through which TRPV4 contributes to disease pathogenesis in conditions such as inflammation, neuropathic diseases (e.g., Alzheimer’s, Charcot- Marie-Tooth disease type 2C), and specific cellular and molecular pathways involving TRPV4, providing insights into its multifaceted involvement in these disorders. TRPV4’s naturally occurring agonist bisandrographolide A was first purified from the plant Andrographis paniculata, which are used in traditional medicine in various parts of Asia for ailments such as upper respiratory tract infections, diarrhea, fever, tonsillitis, and snakebites. In vitro experiments have shown that bisandrographolide A specifically and strongly activates TRPV4, with an EC50 of approximately 800–900 nM, while other TRPV channels show no response at saturation concentrations against TRPV4 [116]. To the best of our knowledge, there is no direct evidence linking these traditional uses to TRPV4 activation. So far, most of the diseases related to TRPV4 mutations are gain-of-function, which makes the TRPV4 antagonism more reasonable treatment for these diseases. Animal studies indicated that many TRPV4 antagonists showed minimal adverse effects [117]. As so far, the first human trial of TRPV4 antagonist GSK2798745 has completed its phase I trial (clinicaltrials.gov, NCT02119260), and the initial findings were promising and showed no significant safety concerns and serious adverse events related to this compound in healthy populations and patients with heart failure [118]. Another study conducted by Valerie J. Ludbrook et al. showed GSK2798745 has no anti-tussive effect in patients with chronic cough and it actually increases ~ 30% in cough counts after a week of GSK2798745 application compared to placebo, and these paradoxical findings suggest that TRPV4 antagonists are involved in a much more complex process than previously thought. Two additional clinical studies related to TRPV4 antagonists have been completed according to the clinical trials database. The first, study NCT02497937, aimed to assess the impact of the TRPV4 blocker GSK2798745 on pulmonary gas transfer and respiration in patients with congestive heart failure and the second trial, NCT02135861, sought to validate a novel treatment for pulmonary edema in cardiac failure. Despite the completion of these trials, there are currently no publications analyzing the results of either study. In animal experiments, the TRPV4 antagonist, HC067047, protected against the fibrosis that develops following alkali burn injury [118, 119]. As previously discussed, the heightened calcium levels led to increase expression and release of proinflammatory cytokines, Thomas Dalsgaard et al. employed GSK2198745, a specific TRPV4 antagonist, demonstrating its protective effect in mice against the lethality induced by lipopolysaccharide (LPS) and cecal-ligation-and-puncture (CLP), but not TNFα, while the blockade of TRPV4 channels significantly reduced the concentrations of pro- inflammatory cytokines, including TNFα, IL-1α, and IL-6. This suggests that the protective effect of TRPV4 may be achieved by inhibiting overwhelming inflammation caused by over-activated TRPV4 [118]. Research findings from animal studies suggest that targeting TRPV4 antagonisms could offer therapeutic benefits for various conditions, including gastrointestinal pain and lung diseases such as cough, pulmonary edema, bronchoconstriction, pulmonary hypertension, and acute lung injury. Despite inconsistencies in observed side effects in vivo, and the complex role of TRPV4 in human diseases, this undoubtedly opens up a challenging new avenue for research into a range of TRPV4-related disorders and the development of TRPV4 modality-specific drugs to treat these conditions.

## Data Availability

No datasets were generated or analysed during the current study.
